# Autochthonous *Angiostrongylus cantonensis*, *Angiostrongylus vasorum* and *Aelurostrongylus abstrusus* infections in native terrestrial gastropods from the Macaronesian Archipelago of Spain

**DOI:** 10.1007/s00436-021-07203-x

**Published:** 2021-06-28

**Authors:** Lisa Segeritz, Alejandro Cardona, Anja Taubert, Carlos Hermosilla, Antonio Ruiz

**Affiliations:** 1 Institute of Parasitology, Biomedical Research Center Seltersberg (BFS), Justus Liebig University Giessen, 35392 Giessen, Germany; 2 Parasitology Laboratory Unit, Faculty of Veterinary Medicine, University of Las Palmas de Gran Canaria, Arucas, Las Palmas, Spain

**Keywords:** Gastropod-borne disease, Lungworm infections, *Angiostrongylus vasorum*, *Angiostrongylus cantonensis*, *Aelurostronglyus abstrusus*, Metastrongyloid nematodes

## Abstract

The presence of zoonotic relevant *Angiostrongylus cantonensis* infections has recently been reported in rat final hosts and gastropod intermediate hosts in Tenerife, Spain. However, data on *A. cantonensis*, *Angiostrongylus vasorum* and *Aelurostrongylus abstrusus* prevalences in endemic gastropods for other islands of the Macaronesian Archipelago are still missing. In order to fill this gap, we conducted an epidemiological study on terrestrial native slug (*Plutonia lamarckii*) and snail (*Cornu aspersum*, *Theba pisana*, *Rumina decollata*) species in 27 selected locations of Tenerife, Gran Canaria, El Hierro, Lanzarote, La Palma and Fuerteventura. Overall, 131 terrestrial gastropods were collected in winter/spring season 2018/2019 and examined for the presence of metastrongyloid lungworm larvae via artificial digestion. The current data revealed a total prevalence of 4.6% for *A. vasorum*, 3.8% for *A. abstrusus* and 0.8% for *A. cantonensis*. In Tenerife, three lungworm species were detected, thereby re-confirming *A. cantonensis* endemicity for this island. Prevalences of snails (*C. aspersum*) originating from El Hierro were 5% for *A. abstrusus* and 15% for *A. vasorum*, respectively, with larval burdens up to 290 larvae per specimen. This epidemiological study indicates the presence of human, canine and feline lungworm species in Macaronesia, Spain. The current data—particularly those on anthropozoonotic *A. cantonensis*—call for a regular large-scale monitoring on intermediate hosts, paratenic hosts and definitive hosts to prevent further spread of lungworm-related diseases in humans and animals.

## Introduction

*Angiostrongylus cantonensis*, *Angiostrongylus vasorum* and *Aelurostrongylus abstrusus* represent lungworms of the family Metastrongyloidea, which can infect humans, domestic dogs and cats. Recent European surveys indicate that these parasites are spreading within Europe (Foronda et al. 2010; Jefferies et al. 2010a; Schnyder et al. 2017; Penagos-Tabares et al. 2020; Federspiel et al. 2020). The zoonotic parasite *A. cantonensis* is found in various rat final host species of Australia, China, India, Pakistan, vast areas of Southeast Asia, Pacific and Indian Ocean islands and South America. It is also endemic in Egypt, the Caribbean area and the southern tropical part of the USA. More recently, *A. cantonensis* has been reported from rats (*Rattus rattus*) (Foronda et al. 2010) and terrestrial gastropods (Martin-Alonso et al. 2011, 2015) of the island Tenerife, Macaronesian Archipelago, and additionally from two hedgehogs of the island Mallorca, Spain (Paredes-Esquivel et al. 2019), proving its geographic expansion into the previously non-endemic European regions (Federspiel et al. 2020). Neurotropic third-stage larvae (L3) of *A. cantonensis* are the etiological agent of human angiostrongyliasis, which is characterized by severe eosinophilic meningoencephalitis/encephalitis with sometime lethal outcome (Martin-Alonso et al. 2015; Federspiel et al. 2020). Humans become infected by oral ingestion of raw or undercooked terrestrial gastropods (i.e. slugs/snails) containing infective L3 or by consumption of undercooked paratenic hosts (i.e. amphibians, crabs, crayfishes). In rats, ingested L3 rapidly migrate through intestinal walls, spread haematogenously, reach cerebrum and cerebellum within 4–6 days *post infectionem* (p. i.), and moult into fourth-stage larvae (L4) which invade the subarachnoid space and migrate via blood circulation to the pulmonary artery and right heart. Conversely, in *A. cantonensis*-infected humans, neurotropic L4 can reach 1–2 mm in size and are often enclosed by granulomas in parenchyma of cerebrum, cerebellum or in the subarachnoid space, thereby causing severe tissue inflammation (Graeff-Teixeira et al. 2009; Wang et al. 2012; Barratt et al. 2016).

In contrast to *A. cantonensis*, the closely related parasite *A. vasorum* is the causative agent of cardiopulmonary disorders of domestic and wild canid species (Taubert et al. 2009; Traversa et al. 2010; Schnyder et al. 2017; Gillis-Germitsch et al. 2017b; Penagos-Tabares et al. 2018a, b; Lange et al. 2018a). As such, adult *A. vasorum* nematodes parasitize the right heart and pulmonary artery system of dogs (Schnyder et al. 2017; Penagos-Tabares et al. 2018a), wolves (Eleni et al. 2014; Hermosilla et al. 2017), coyotes (Bourque et al. 2005), jackals (Takács et al. 2014) and several fox species (Lima et al. 1994; Duarte et al. 2007; Morgan et al. 2008; Schug et al. 2018). Additionally, mustelids (e.g. ferrets, racoon dogs) and other carnivorous species may act as definitive hosts (Lemming et al. 2020). Canine angiostrongylosis might vary from subclinical cases to ophthalmic, nervous and severe cardiopulmonary disorders including life-threatening systemic coagulopathies (Taubert et al. 2009; Di Cesare and Traversa 2014; Schnyder et al. 2017).

*Aelurostrongylus abstrusus* is a relevant lungworm of domestic and wild felids with a worldwide distribution (Scott 1973; Traversa et al. 2008; Taubert et al. 2009; Jefferies et al. 2010b; Elsheikha et al. 2019). Clinical manifestations of feline aelurostrongylosis include typical signs of respiratory diseases, such as dyspnoea, mucopurulent nasal discharge, open-mouthed abdominal breathing, sneezing, coughing and wheezing (Traversa et al. 2008). *Aelurostrongylus abstrusus* was reported in various European (Traversa et al. 2008; Taubert et al. 2009; Jefferies et al. 2010b; Knaus et al. 2011) and South American (Penagos-Tabares et al. 2018a) countries and is infecting, besides domestic cats, also several felid wildlife species (West et al. 1977; Noronha et al. 2002; González et al. 2007; Penagos-Tabares et al. 2018a).

The lungworms *A. cantonensis*, *A. vasorum* and *A. abstrusus* have been considered neglected and underestimated in Europe (Foronda et al. 2010; Martin-Alonso et al. 2011, 2015; Lange et al. 2018a) and other geographic regions (Traversa et al. 2010; Penagos-Tabares et al. 2018a, 2019; Federspiel et al. 2020). Nowadays, due to an increased research focus on lungworms, occurrences of these nematodes are reported from several geographical areas of Europe (Morgan and Shaw 2010; Taylor et al. 2015; Barutzki et al. 2017; Maksimov et al. 2017). Since metastrongyloid lungworms require terrestrial gastropods as obligate intermediate hosts to complete their life cycles, knowledge on seasonal infections in gastropods seems crucial for a better understanding of the epidemiology of these parasitoses. Whilst a vast amount of data exists on lungworm infections in definitive hosts (Taubert et al. 2009; Wang et al. 2012; Barutzki and Schaper 2013; Di Cesare et al. 2015; Barratt et al. 2016; Gillis-Germitsch et al. 2017a; Schnyder et al. 2017), little has been reported on natural gastropod infections (Ferdushy et al. 2009; Majoros et al. 2010; Patel et al. 2014; Lange et al. 2018a, b; Penagos-Tabares et al. 2019, 2020; Dimzas et al. 2020). To our best knowledge, there is only one report on *A. cantonensis*-infected slugs/snails in Tenerife (Martin-Alonso et al. 2015).

Therefore, the aim of this study was firstly to evaluate lungworm larval infections in native terrestrial gastropod populations, and secondly, to address a potential geographic expansion of these parasites into other islands of the Macaronesian Archipelago in Spain.

## Materials and methods

### Study area and slug/snail collection

**Fig. 1:**
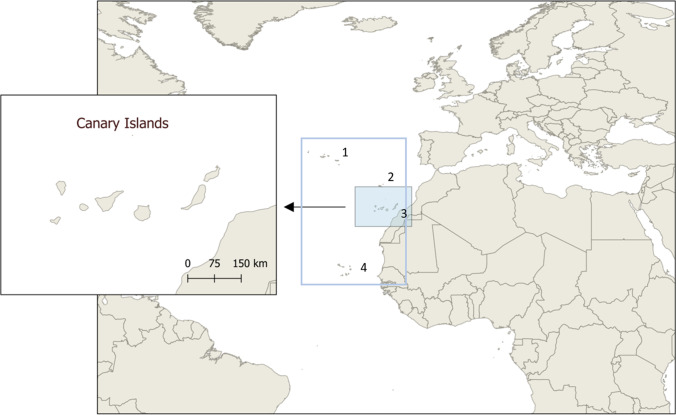
The Macaronesian region: (1) Azores; (2) Madeira; (3) Canary Islands; (4) Cape Verde

As also true for the Spanish mainland, a temperate Mediterranean climate predominates in the Archipelago of Macaronesia (see Fig. 1). Rainfall can be scarce in distinct islands (i.e. Lanzarote and Fuerteventura), where prolonged dry seasons and hot summers are typical. Each island has an individual microclimate, based on a distinct geography and eco-epidemiology, including endemic vegetation and vertebrate/invertebrate species diversity. The current terrestrial mollusc collection sites were diverse but semi-arid climate conditions were predominant (see Table 1). Thus, collection sites were mainly composed of xerophyts (e.g. cactus and euphorbia), grassland, shrubs, and also sub-tropical pine and laurel forests (in Tenerife, El Hierro and La Palma) with evergreen endemic trees, such as *Pinus canariensis* and broadleaf Lauraceae (e.g. *Laurus novocanariensis* and *Persea indica*).

**Table 1:** Characteristics of sampling locations, with corresponding numbers of collected and lungworm-positive detected gastropods from Macaronesia, Spain

Location	Municipality	Vegetation	Climate*	Zone	Collected gastropods	Lungworm-positive gastropods
Gran Canaria						
Lomo Apolinario	Las Palmas de Gran Canaria	Sweet spurge shrubland	BWh	Urban	13	0
Altos Guía	Santa María de Guía	Sweet spurge shrubland	Csc	Rural	5	0
Maspalomas	San Bartolomé de Tirajana	Hyper-arid tamarisk thicket	BWh	Urban	2	0
Firgas	Firgas	Laurisilva	BSk	Rural	3	0
Tenerife						
Santa Cruz de Tenerife	Santa Cruz de Tenerife	Cardon spurge shrubland	BSh	Urban	5	0
Tegueste	Tegueste	Laurisilva	Cs	Rural	5	0
La Esperanza	El Rosario	Laurisilva	Cs	Rural	5	4
Guía de Isora	Guía de Isora	Pine forest	Csb	Rural	2	1
La Orotava	La Orotava	Laurisilva	Csb	Suburban	4	1
Icod de los Vinos	Icod de los Vinos	Pine forest	BSh	Rural	4	0
San Cristóbal de la Laguna	San Cristóbal de la Laguna	Laurisilva	Csb	Urban	3	0
Lanzarote						
Puerto del Carmen	Tías	Sweet spurge shrubland	BWh	Urban	10	0
Arrieta	Haría	Sweet spurge shrubland	BWh	Suburban	10	0
Fuerteventura						
La Oliva	La Oliva	Cardon spurge shrubland	BWh	Rural	10	0
Corralejo	La Oliva	Psammophilous communities	BWh	Rural	10	0
La Palma						
Fuencaliente	Fuencaliente	Pine forest	Csb	Rural	2	0
Tijarafe	Tijarafe	Pine forest	Csb	Rural	2	0
Los Llanos de Aridane	Los Llanos de Aridane	White broom shrubland	Csa	Urban	2	0
Puntagorda	Puntagorda	Pine forest	Csb	Rural	2	0
Santa Cruz de la Palma	Santa Cruz de la Palma	Cardon spurge shrubland	BSh	Urban	6	0
Barlovento	Barlovento	Laurisilva	Csa	Rural	6	0
El Hierro						
La Frontera	La Frontera	Juniper woodland	BSh	Rural	1	0
Isora	Valverde	Pine forest	BSk	Rural	2	0
Moncanal	Valverde	Laurisilva	BSk	Rural	3	0
San Andres	Valverde	Laurisilva	BSk	Rural	5	0
Sabinosa	La Frontera	Juniper woodland	BSk	Rural	3	0
Valverde	Valverde	Laurisilva	BSk	Rural	3	3
Ruta del Garoé	Valverde	Laurisilva	BSk	Rural	3	0

*Köppen-Geiger climate classification:

*BSk*, cold semi-arid climate; *BSh*, hot semi-arid climate; *BWh*, hot desert climate; *Cs*, Mediterranean climate; *Csa*, hot-summer Mediterranean climate; *Csb*, warm-summer Mediterranean climate; *Csc*, cold-summer Mediterranean climate

All climate data according to www.climate-data.org

All data about vegetation according to the book of Marcelino J. del Arco Aguilar and Octavio Rodríguez Delgado (2018) Vegetation of the Canary Islands

Study areas included locations, where straying dogs, feral cats, black rats (*Rattus rattus*) and brown rats (*Rattus norvegicus*) were frequently present, since these mammals can potentially act as definitive hosts for human and canine angiostrongylosis as well as feline aelurostrongylosis/troglostrongylosis.

**Fig. 2:**
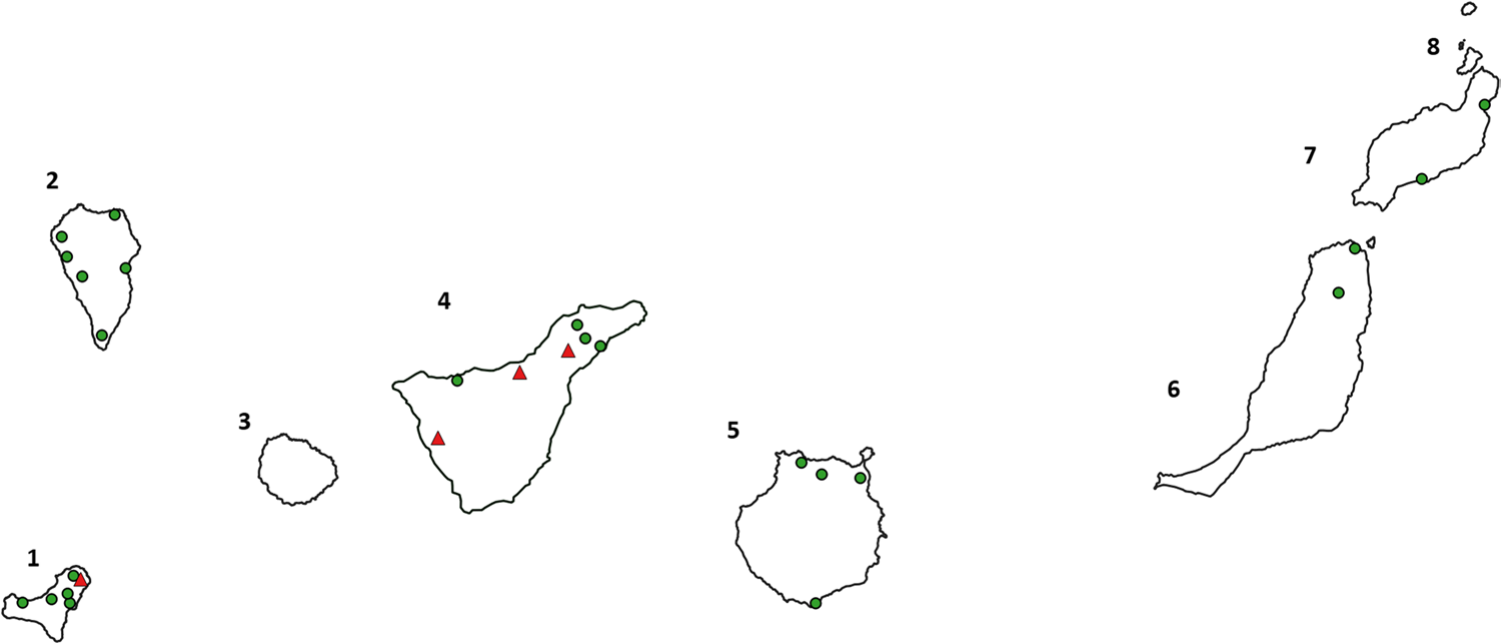
Sampling locations. Green circle means locations with lungworm negative gastropod samples. Red triangle means locations with lungworm-positive gastropod samples. (1) El Hierro: La Frontera; Isora; Mocanal; San Andrés; Sabinosa; Valverde; Ruta del Goroé. (2) La Palma: Fuencaliente; Tijarafe; Los Llanos de Aridane; Puntagorda; Santa Cruz de la Palma; Barlovento (3) La Gomera (4) Tenerife: Santa Cruz de Tenerife; Tegueste; La Esperanza; Guía de Isora; La Orotava; Icod de los Vinos; La Laguna (5) Gran Canaria: Las Palmas de Gran Canaria; Santa María de Guía; Maspalomas; Firgas (6) Fuerteventura: La Oliva; Corralejo (7) Lanzarote: Puerto del Carmen; Arrieta (8) La Graciosa

Different native terrestrial gastropods, such as slugs, snails (*Theba pisana*, *Cornu aspersum*, *Rumina decollata*) and the so-called semi-slugs (*Plutonia lamarckii*), which are characterized by the appearance of slugs but are equipped with a small rudimental shell, were collected. Gastropod collection occurred mainly by hand at dawn or break of dawn. In total, 131 specimens were collected during December 2018 to April 2019 from Tenerife (*n* = 28), Gran Canaria (*n* = 23), Lanzarote (*n* = 20), El Hierro (*n* = 20) and Fuerteventura (*n* = 20), Archipelago of Macaronesia, Spain (24° 15′ 24″ N, 22° 28′ 16″ W; see Fig. 1 and Fig. 2). Furthermore, we also intensively searched on La Gomera, but failed to find gastropods.

Molluscs were weighted, individually stored in plastics bags, cryo-euthanized according to Lange et al. (2017) and stored frozen at − 20 °C at the Faculty of Veterinary Medicine, University of Las Palmas de Gran Canaria, Spain, until further investigations.

Before artificial digestion, gastropod species were identified based on their morphological characteristics according to literature (Nordsieck 2000; Martin-Alonso et al. 2015). Frozen gastropods were transferred to the Institute of Parasitology at Justus Liebig University Giessen (JLU), Giessen, Germany. According to current national animal protection laws of Spain, a permission for gastropod collection or their use for basic research purposes is not required.

### Gastropod digestion

Frozen gastropods (*P. lamarckii*, *C. aspersum*, *T. pisana*, *R. decollata*) were cut into small pieces and immersed in digestion solution [10 g pepsin powder 2000 FIP-U/g (Robert Kind), 8.5 g NaCl (Carl Roth), 30 mL HCl 37% (Carl Roth), adjusted with distilled water to 1 L] according to Lange et al. (2018a, b) and Penagos-Tabares et al. (2020). Briefly, single gastropods were digested for 3 h at 37 °C in sterile 50-mL plastic tubes (Greiner) under constant shaking conditions. Digested gastropod samples were sieved according to Lange et al. (2018a, b) through a 300-µm metal sieve (Retsch, Haan, Germany) in order to remove any un-digested material, and afterwards passed through a 25-µm metal sieve (Retsch, Haan, Germany).

### Morphological and morphometric identification of metastrongyloid larvae

**Fig. 3:**
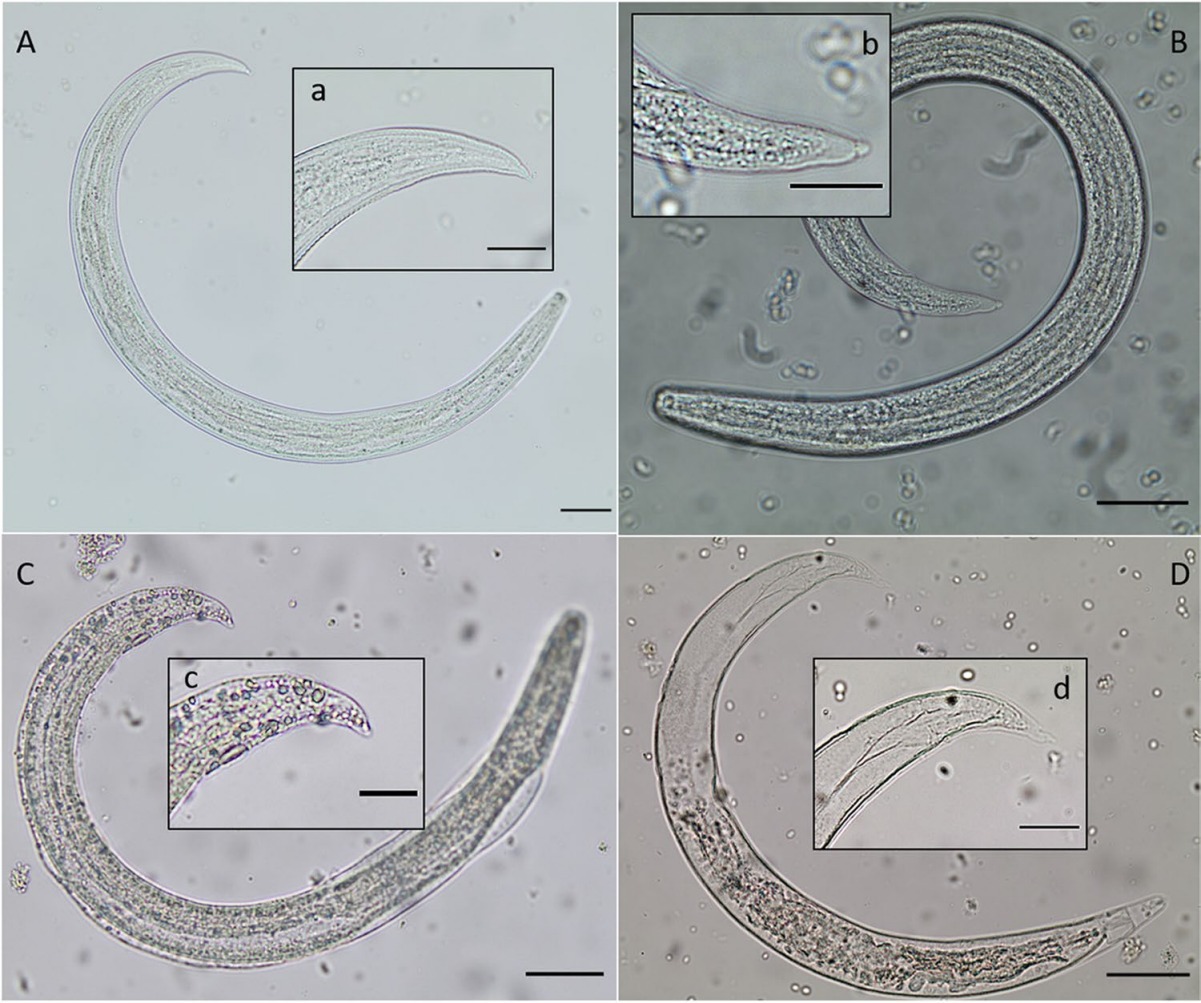
Morphological characteristics of metastrongyloid larvae, found in gastropods from Macaronesia, Spain. Third-stage larvae of *Angiostrongylus cantonensis* (A), *Aelurostrongylus abstrusus* (B) and *Angiostrongylus vasorum* (C); panel D shows an *A. vasorum* second-stage larva, in the sheath of a first-stage larva; details of the posterior ends are shown in (a), (b), (c) and (d). The third-stage larva of *A. cantonensis* can be identified by its tail pointed tip (a), whereas the *A. abstrusus* L3 (b) has a terminal rounded knob and the *A. vasorum* L3 is characterized by a short digitiform posterior end (c). The tail of an *A. vasorum* L1 shows a dorsal spine (d). Scale bar (A, B, C, D) 40 μm; scale bar (a, b, c, d) 20 μm

Remnants of the last sieving process were transferred into sterile 15-mL Falcon tubes and pelleted at 40 × *g* for 10 min at room temperature (RT). Pellets were re-suspended and examined via an Olympus BH-2® microscope equipped with a digital camera (SC30®, Olympus). Metastrongyloid species and stages were identified morphologically, documented individually by digital photography. Larvae were counted, carefully collected by pipetting under microscopic control and stored at 4 °C for further examinations. The body length and width, the oesophagus form (non-rhabditiform), the ratio of oesophagus to body lengths (1:3–1:2) and the typical larval tail morphology were analysed as reported elsewhere (Lange et al. 2018a; Guilhon and Cens 1973; Giannelli et al. 2014; Martin-Alonso et al. 2015; Penagos-Tabares et al. 2018a) (see Fig. 3).

### Molecular identification

To confirm morphological findings, DNA from pooled metastrongyloid larvae of single gastropods was isolated using a commercial kit (Quiagen DNeasy Blood and Tissue Kit®) and analysed as described previously (Lange et al. 2018a, b; Penagos-Tabares et al. 2019, 2020). Molecular species confirmation was performed by running conventional PCRs with the universal nematode primers NC1/NC2 (Gasser et al. 1993) followed by species-specific real-time PCRs. A duplex real-time PCR for *A*. *abstrusus* and *T*. *brevior* was carried out, amplifying the internal transcribed spacer 2 (ITS-2) region from the ribosomal DNA. This PCR was conducted, using the forward primers TrogloF and AeluroF and the single reverse primer MetR (Annoscia et al. 2014). The duplex real-time PCR for *A*. *vasorum* was performed, amplifying a partial ITS-2 region as reported by Jefferies et al. (2009).

## Results

### Prevalence of *A. cantonensis*, *A. vasorum* and *A. aelurostrongylus* in native gastropods

Considering all samples from all regions, a total lungworm larvae prevalence of 6.9% (9/131) with a 95% confidence interval (CI) of 2.6–11.2 was calculated, based on microscopic identification. Lungworm larvae were present in gastropods from two out of six investigated islands (see Fig. 2 and Table 2), giving a total prevalence of 21.4% (6/28) (6.2–36.6 CI 95%) in Tenerife and of 15% (3/20) (0–30.6 CI 95%) in El Hierro. In Tenerife, three lungworm-positive gastropod locations were identified, whereas in El Hierro only one collection site proved positive for lungworm larvae in gastropods, namely the municipality of Valverde.

**Table 2:** Prevalence of lungworm species in natural intermediate host populations in Macaronesia

Lungworm species	Total P (+ /n) [95%CI]	Tenerife P (+ /n) [95%CI]	El Hierro P (+ /n) [95%CI]
All lungworm species	6.9 (9/131) [2.6–11.2]	21.4 (6/28) [6.2 –36.6]	15 (3/20) [0–30.6]
*Angiostrongylus vasorum*	4.6 (6/131) [1–8.2]	10.7 (3/28) [0–22.1]	15 (3/20) [0–30.6]
*Aelurostongylus abstrusus*	3.8 (5/131) [0.5 –7.1]	14.3 (4/28) [1.3 –27.3]	5 (1/20) [0–14.6]
*Angiostrongylus cantonensis*	0.8 (1/131) [0–2.3]	3.6 (1/28) [0–10.5]	0 (0/20) [0]

*P*, prevalence in %; *n*, number of analysed gastropods; + , number of positive samples; *CI*, confidence interva

The most common parasite was *A. vasorum* with a prevalence of 4.6% (6/131; 1–8.2 CI 95%) and *A. abstrusus* with a prevalence of 3.8% (5/131; 0.5–7.1 CI 95%), followed by *A. cantonensis* (0.8%; 1/131; 0–2.3 CI 95%) (see Table 2).

Unfortunately, the samples did not contain sufficient amplifiable DNA for molecular analyses. Therefore, for prevalence calculation, only microscopic-based data was used.

### Larval stages and burden in gastropods

A high proportion of lungworm larvae in gastropod samples revealed as second-stage (L2, 50.3%) or third-stage (L3, 46.2%) larvae, whilst only 3.5% of the larvae were L1 (see Table 3). Of note, all white garden snails (*T. pisana*; *n* = 51) proved negative for lungworm larvae. Thus, lungworm larvae were exclusively found in native semi-slugs [*P. lamarckii* (2/5)], common garden snails [*C. aspersum* (5/63)], decollate snails [*R. decollata* (1/5)] and one unidentified slug species (1/7).

**Table 3:** Metastrongyloid-positive samples with the identified lungworm species, the number of detected larval stages, the larval burden and information on the gastropod species and the location, vegetation and climate where the sample originates from

Location	Vegetation	Climate*	Gastropod species	Detected lungworm species	Detected larval stages				Larval burden
					L1	L2	L3	Unknown	
Tenerife									
La Esperanza	Laurisilva	Csb	*R. decollata*	*A. abstrusus*	2	6	1		9
La Esperanza	Laurisilva	Csb	*C. aspersum*	*A. abstrusus*	0	1	0		1
La Esperanza	Laurisilva	Csb	*P. lamarckii*	*A. abstrusus*	0	1	32		33
La Esperanza	Laurisilva	Csb	*P. lamarckii*	*A. abstrusus* *A. vasorum*	0	41	97		138
Guia de Isora	Pine forest	Csb	Slug**	*A. vasorum*	0	4	1		5
La Orotava	Laurisilva	Csb	*C. aspersum*	*A. cantonensis* *A. vasorum*	3	12	23		38
El Hierro									
Valverde	Laurisilva	BSk	*C. aspersum*	*A. abstrusus* *A. vasorum*	11	159	56	64	290
Valverde	Laurisilva	BSk	*C. aspersum*	*A. vasorum*	0	2	0		2
Valverde	Laurisilva	BSk	*C. aspersum*	*A. vasorum*	0	3	0		3
				Total	16	229	210	64	519
				Mean	5.3	25.4	35.0		57.7
				Distribution in %	3.5	50.3	46.2		

*Köppen-Geiger climate classification:

*Csb*, warm-summer Mediterranean climate; *BSk*, cold semi-arid climate

All climate data according to www.climate-data.org

**Unidentified species.

Larval burden per specimen varied considerably from one to 290 larvae for individual gastropods. Overall, 55.6% (5/9) of metastrongyloid-positive gastropods contained less than 10 larvae. Interestingly, the two molluscs (*P. lamarckii* and *C. aspersum*) with the highest larval burden (138 and 290 larvae, respectively) both showed a double infection with *A. vasorum* and *A. abstrusus*. Coinfections were observed in three gastropods. One gastropod (*C. aspersum*) contained larvae, which could not be identified due to their destroyed cuticles, oesophagus or tails. Besides lungworm larvae, some typical gastropod-specific parasitic nematodes of the genus *Phasmarhabditis* were detected, but not further considered in this study.

### Discussion and conclusion

The present epidemiological study offers conclusive evidence that native gastropod species in Macaronesia may act as suitable intermediate hosts for human and canine angiostrongylosis and feline aelurostrongylosis, under natural habitat conditions. It further represents the first report in Spain on *A. vasorum* and *A. abstrusus* infections, occurring in terrestrial intermediate hosts, thereby underlining their role in the epidemiology of these neglected parasitoses. It also confirms recent reports on the occurrence of anthropozoonotic-relevant *A. cantonensis* in Tenerife. The biodiversity of terrestrial gastropod species in this particular region of Spain gives evidence that native molluscs will most probably contribute to expansion of zoonotic- and veterinary-relevant lungworms into other islands where infections of humans, dogs and cats with these lungworms have not yet been reported.

The distribution pattern of metastrongyloid-positive gastropods in Tenerife indicated a widespread geographical extension of these parasites on this island. Whilst other related studies described an occurrence of *A. cantonensis* in the northeastern part of the island (Foronda et al. 2010; Martin-Alonso et al. 2011, 2015), here we detected *A. cantonensis* infections in the municipality Guia de Isora, which is situated in the western part of Tenerife. *A. cantonensis* infections remained restricted to this island, thereby confirming its geographic endemicity (Foronda et al. 2010; Martin-Alonso et al. 2011, 2015), but denying further expansion into other archipelago islands.

Since the Canary Archipelago has a good infrastructure and is popular for tourism, an oversea transportation system by ferries is operated between the islands; thereby, facilitating transmission of lungworm-infected definitive hosts is more likely. Accordingly, we expected to detect more lungworm-positive islands/locations. In Gran Canaria, the second most populated and a well-travelled island of the Canary Archipelago, aelurostrongylosis was diagnosed 2016 for the first time in feral cats (Rodriguez-Ponce et al. 2016). No *A. abstrusus*-infected gastropods were found in the current study on this island. One explanation for this result may be the limited number of analysed molluscs. The presence of gastropods and their parasite burden seems to be dependent on different climatic, ecological and environmental factors (Lv et al. 2006; Ferdushy et al. 2010; Giannelli et al. 2016). Furthermore, individual and species-specific coprophilic/coprophagic behaviour in gastropods plays a role in the intermediate host capability to become infected by lungworm larvae. As gastropods are humidity dependent, finding them, in locations with vast semi-arid areas and valleys, as observed in Gran Canaria, is challenging. In the relatively humid island of the Canary Archipelago, La Gomera, which represents an apparently good habitat for gastropods, we expected to find a richer mollusc population. However, on this island, no gastropods were found despite two excursion efforts into different geographic locations.

The current data showed both lungworm coinfections and high parasitic burden in single Macaronesian gastropods. Thus, approximately half of the infected gastropods (5/9) comprised more than one lungworm species. Assuming that a longer exposure to faeces, covering metastrongyloid larvae leads to a higher probability for gastropods to become infected, we also expect that the occurrence of coinfections and the larval burden are dependent on this parameter. Besides age, also coprophagic preferences in oral uptake influence gastropod’s exposure to infective L1. Therefore, factors affecting coprophagic behaviour in gastropods should be addressed in future studies. All here investigated metastrongyloid lungworms show a rather broad intermediate host spectrum, implicating that multiple intermediate hosts might exist at current sampling sites. Further studies on complex parasite-intermediate host interactions are currently planned to better understand gastropod-derived innate immune reactions against these larval stages. Consistently, it was recently demonstrated that gastropod-derived haemocytes seem capable to rapidly cast the so-called invertebrate extracellular phagocyte traps (InEPTs), mainly composed of extruded chromatin decorated with anti-microbial components, not only against motile larval stages of *A. vasorum* but also against *A. abstrusus* (Lange et al. 2017; Penagos-Tabares et al. 2018b), thereby demonstrating similarities to mammalian-derived neutrophil extracellular traps (NETs) against parasites (Silva et al. 2016; Villagra-Blanco et al. 2019). Since DNA isolation of a single larva is challenging and some detected larvae of the current study were in very poor conditions, DNA amplifying failed. Furthermore, this might be linked, besides to the DNA inhibitory effects deriving from snail tissue, to InEPTs-derived effector mechanisms including degranulation, reactive oxygen species (ROS) generation and encapsulation in vivo, resulting in partial or complete larval degradation (Penagos-Tabares et al. 2018b). Consequently, early host innate immunity and biodiversity of endemic intermediate gastropod hosts should be taken into account to better understand biology, distribution and expansion of human and canine/feline lungworms within the Macaronesian Archipelago and elsewhere. Considering the unique endemic vegetation and specific fauna as well as the local situation with high populations of feral dogs, cats and rats (Nogales et al. 2004; Medina and Nogales 2009; Martin-Alonso et al. 2011, 2015; Rodriguez-Ponce et al. 2016; Carretón et al. 2020), large-scale epidemiological surveys are needed, addressing definitive hosts (dogs, cats, rats) and gastropods as well as paratenic hosts (birds, reptiles, rodents) within this archipelago. Preventive education on this neglected parasitosis as well as food inspections is necessary, as snail consumption by humans is a common practice in Macaronesia. Thus, this work aims to raise awareness of veterinary surgeons, physicians and public health authorities not only in Macaronesia but also in other regions with similar climatic conditions.
